# Using Discharge Abstracts to Evaluate a Regional Perinatal Network: Assessment of the Linkage Procedure of Anonymous Data

**DOI:** 10.1155/2009/181842

**Published:** 2008-12-23

**Authors:** Catherine Quantin, Béatrice Gouyon, Paul Avillach, Cyril Ferdynus, Paul Sagot, Jean-Bernard Gouyon

**Affiliations:** ^1^INSERM, UMR 866, University of Burgundy, 21000 Dijon, France; ^2^Division of Medical Informatics, University Hospital, 21000 Dijon, France; ^3^Laboratoire d'Enseignement et de Recherche sur le Traitement de l'Information Médicale (LERTIM), Faculté de Médecine, Université de la Méditerranée, 13385 Marseille, France; ^4^Institut de Santé Publique, d'Epidémiologie et de Développement (ISPED), Université Victor Segalen Bordeaux II, 33076 Bordeaux, France; ^5^Division of Obstetrics and Gynaecology, University Hospital, 21079 Dijon, France; ^6^Division of Pediatrics, University Hospital, 21079 Dijon, France

## Abstract

To assess the Burgundy perinatal network (18 obstetrical units; 18 500 births per year), discharge abstracts and additional data were collected for all mothers and newborns. In accordance with French law, data were rendered anonymous before statistical analysis, and were linked to patients using a specific procedure. This procedure allowed data concerning each mother to be linked to those for her newborn(s). This study showed that all mothers and newborns were included in the regional database; the data for all mothers were linked to those for their infant(s) in all cases. Additional data (gestational age) were obtained for 99.9% of newborns.

## 1. Introduction

The Burgundy perinatal network was created in 1992 to improve the quality of perinatal care in Burgundy, a French region with 1 800 000 inhabitants and 18 500 births annually. This network gradually included the 18 private and public hospitals of Burgundy. The medical steering committee, made up of medical representatives of each hospital (one paediatrician and one obstetrician for each) has progressively reorganized perinatal care in the region. In particular, medical conventions between hospitals were signed to improve coordination of admissions, transfers of patients, and the use of technical facilities. Moreover, a half-yearly systematic analysis of perinatal deaths was initiated.

The impact of actions promoted in the perinatal network has been assessed yearly since 1998.

A multidisciplinary working group previously chose and precisely defined specific indicators (25 for each mother and 17 for each newborn) [1, 2]. These indicators correspond to maternal medical history, psychosocial risk factors, maternal and neonatal medical data such as hospitalisations during pregnancy, delivery method, postpartum period, and the different hospitalisations of the newborns. Most of these items could be obtained from the mandatory discharge abstracts for each hospitalised patient within the Programme de Médicalisation du Système d’Information (PMSI). The PMSI collection system was used as it is compulsory in both public and private hospitals in France. Each hospital provides its own perinatal data on a voluntary basis. These data are rendered anonymous before being sent from each hospital to the committee in charge of the assessment of the perinatal network's performance (regional audit committee).

Optimal assessment of perinatal care obviously requires effective linkage between maternal and neonatal data. Indeed, in order to study the mechanisms of neonatal diseases it is essential to link (1) the abstracts of a mother to those of her newborn even if they were not cared for in the same hospital and (2) all discharge abstracts for the same patient (mother and/or newborns), who may have several discharge abstracts from different hospitals. In our case, this happens when an antenatal or postnatal transfer from one hospital to another is decided, according to the written rules for good medical practice in the perinatal network.

This study intends to assess the performance of the file-linkage process of maternal and neonatal data used for the evaluation of the Burgundy perinatal network.

## 2. Materials and Methods

### 2.1. Population

All deliveries and births, whether live or not, are included in the perinatal network if the gestational age is at least 22 weeks and/or if the birth weight is greater than 500 g. For the purpose of this study, we took into account the population included from 1998, the year data collection began, to 2001. In 1998, only nine hospitals participated in the data collection, whereas in 2001 all of the 18 hospitals involved in the regional perinatal care network were involved, thus providing data for 100% of the 18 500 annual births.

### 2.2. Data Collection

Discharge abstracts for all mothers (social and medical data about gestation, childbirth and the postnatal period) and all newborns (hospitalisations in maternity hospitals, paediatric wards, neonatal units, and neonatal intensive care units) collected within the PMSI were provided by each hospital in Burgundy. The collection of discharge abstracts is mandatory and does not require the agreement of the patient. The usual data collection system of the PMSI was expanded to include the collection of birth weight and gestational age. Moreover, six identification items (maiden names, first names and birth dates of mothers, first names and birth dates of newborns, zip codes of the main residence of mothers) were recorded for both mothers and newborns to allow linkage between their discharge abstracts.

### 2.3. Anonymity of Information

The Directive 95/46/CE of the European Parliament and of the Council of 24 October 1995 on the protection of individuals with regard to the processing of personal data and on the free movement of such data and the Act n°78-17 of 6 January 1978 on Data Processing, Data Files and Individual Liberties (amended by the Act of 6 August 2004 relating to the protection of individuals with regard to the processing of personal data) are the European and French legislations on data privacy and security. Following these recommendations, the data were rendered anonymous in each hospital using 3 ANONYMAT software [3–5]. The principle of this software based on the Standard Hash Algorithm is to ensure the irreversible transformation of independent fields, which prevents the identification of an individual. The aim is to obtain a strictly anonymous code, but always the same one for a given couple of a mother and her newborn(s). The use of ANONYMAT software has been authorized by the French Department for Information System Security (SCSSI), and in 1998 the French data protection commission (Commission Nationale de l'Information et des Libertés—CNIL) specifically authorized the management of perinatal data in Burgundy. The collection of the data does not require the specific consent of the patient, who only has to be informed that the information contained in discharge abstracts will be used for epidemiological purposes and not only for budgetary purposes. An information leaflet explaining the use of the data is given to the patient on admission to the hospital.

In order to reduce the impact of typing errors, spelling transformation was introduced into the anonymity process before hash coding. The principle is to transform the spelling of names according to phonetic rules. Among the different treatments, SOUNDEX is most widely used. However, in the perinatal network, a treatment specifically adapted to the particularities of the French language was used [6].

### 2.4. File Linkage

**Figure 1:**
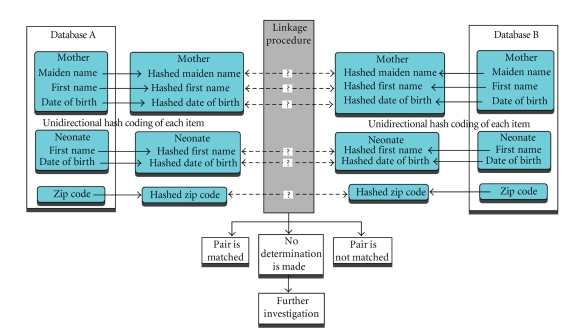
File linkage.

Once rendered anonymous, the data were transmitted to the regional audit committee located in the University Hospital of Dijon (France) and included in a regional database (cf. Figure 1). The first part of the linkage process is to gather all discharge abstracts concerning the same patient (mother or newborns) from the same or different hospitals. The second part is devoted to linking the abstracts of a mother to those of her newborn even if they were not cared for in the same hospital. In order to respect the confidentiality of medical files, linkage is carried out on files that are rendered anonymous and not directly on nominative data. Technically, there is no difference between using original nominative data or one-way hash-coded data for record linkage, as the correspondence between these data is almost one-to-one (low collision rate). In fact, the spelling processing used in the anonymity procedure reduces the impact of typing errors and increases the efficiency of the linkage. However, two types of linkage errors are of concern: erroneous links of notifications (information) from two distinct patients, also called homonym errors, and failure to link multiple notifications (information) on the same patient, also called synonym errors.

These errors would be reduced if we could use the French Identification Number which is unique for each individual. However, this number is not recorded by hospitals, which only use the Social Security Number (SSN), which is not unique for each individual as it is assigned to each insured person, who may register his family under the same SSN. Thus, the SSN of a woman may change, for example, after her marriage. Moreover, communication of the SSN is not allowed by French legislation except for the transmission of information from private-sector hospitals to French health-insurance companies.

Therefore, we have developed a record linkage system which is based on the method proposed by Jaro [7] and takes into account the 6 identification variables.

In this procedure, like in other probabilistic methods [8–15], a weight is associated with each variable according to the reliability of the information provided. The weight given to two identical dates of birth is then higher than that given to two identical zip codes, as date of birth is more discriminating than zip code. It is then possible to compute the weight corresponding to each pair of records by summing the positive weights obtained for concordant variables and negative weights for discordant variables. Two threshold values can then be computed, from which three sets of possible decisions can be determined as follows: (1) the pair is matched; (2) no determination is made; (3) the pair is not matched. In case 2, no determination, other information is explored.

### 2.5. Reliability of the Linkage Procedure

It was assumed that a link between a mother and a noncorresponding newborn was impossible in the case of perfect agreement between two records on the 6 identification items. Indeed, it is highly unlikely that in the same maternity hospital, two women having the same zip code, the same maiden name, the same first name, the same birth date would give birth on the same day to babies with the same first name. Additionally, the risk for a homonym error was very low (10^−48^) with the standard hash algorithm [16].

In a perinatal network, it is obvious that every newborn has a mother and vice versa. When a mother abandons her infant at birth and has the right to remain anonymous, the maiden name, the first name, and the date of birth of the mother were filled with random numbers and thus rendered anonymous in the hospital file before hash coding, both in the mother's and in the newborn's files. As the linkage items for the mother and her newborn are the same (filled in with the same numbers), linkage is possible.

So, the fact that no link was found between a newborn and a mother would indicate either a linkage error or the lack of the mother's record. Linkage errors were identified by again using the linkage method on the basis of only five items or even four. To verify these potential links, each hospital was asked to verify the identification items of the records corresponding to the given anonymous numbers. The corrected data were rendered anonymous before being returned from the hospitals to the regional database.

Finally, the linkage was only performed on the basis of all 6 identification items in the final database.

### 2.6. Data Validation

Before linkage, the exhaustiveness of both medical items and linkage items was monitored as follows: (1) exhaustiveness for the 6 linkage items was verified for each discharge abstract; (2) exhaustiveness for gestational age and birth weight was verified for each neonatal discharge abstract.

After linkage, the exhaustiveness for the number of mothers and newborns registered in the regional database was assessed from hand-written notebooks which are used in each hospital for the registration of births and/or admissions of sick newborns to units caring for these infants. The law requires these hand-written notebooks to be completed.

Regarding data quality, computerized procedures have also been developed to reveal discrepancies between medical data or between dates of exit and admission for successive hospitalisations. These inconsistencies were validated by a paediatrician. All erroneous data were then corrected in the nominative files in each hospital, before being rendered anonymous and sent back to the regional database. As a consequence, after validation, the number of mothers and newborns agreed with hand-written notebooks, and the medical or administrative inconsistencies between discharge abstracts were corrected. We then considered data after validation as a gold standard for the assessment of the linkage procedure (on data before validation).

## 3. Results

This collection started in 1998. The exhaustiveness of data collection and the linkage rate have improved with time.

During validation after linkage, the number of mothers and newborns registered in the regional database was assessed from the hand-written notebooks which are used in each hospital for the registration of births and/or admissions of sick newborns to units caring for these infants. At the beginning of the collection in 1998 or 1999, we detected some discrepancies. We thus had to modify the selection criteria for data collection. From 2000, the consistency between these numbers was perfect.

**(1) Exhaustiveness of Discharge Abstracts**

In 1998, 9 hospitals were involved in the collection of discharge abstracts. Abstracts for 84.1% and 99.1% of all eligible mothers were retrieved in the regional database, respectively, before and after the validation procedure; for newborns, the percentages were 100% and 98.7%. Overestimation of the hospitalisation rate in some neonatal files in 1998 was explained by undue inclusion of hospitalisation beyond the neonatal period.

From 2001 to 2005, 18 hospitals were involved in the collection of discharge abstracts and the overall exhaustiveness for both mothers and newborns reached 99.9% after the validation procedure. The exhaustiveness of discharge abstracts was 100% but some mothers may not have been hospitalised if the delivery occurred at home.

**(2) Exhaustiveness of the Data in the Discharge Abstracts**

In 1998, the six items used for the linkage procedure were recorded in 80% of discharge abstracts before validation and in 99% after validation. From 2001 to 2005, the exhaustiveness of these items was between 91.9% and 98.5% before and 100% after validation.

**(3) Linkage Assessment**

Before validation, the percentage of newborns linked to their corresponding mothers on the basis of the 6 identification items was 71% in 1998, 92.9% in 2001, and 93.4% in 2005.

Different types of errors were found during the validation procedure after linkage. The most frequent one corresponded to errors in spelling surnames and first names [6] leading to phonetic changes that were not subsequently corrected by the spelling processing included in the ANONYMAT software. The inversion of the married name, and the maiden name, during data collection was also a source of linkage failure, and was corrected through the validation procedure. After validation, 99.98% of newborns were linked to their mothers whatever the year concerned. Only newborns who were transferred from a hospital of the Burgundy Region to a hospital of another region were missed (0.2%).

The results regarding the assessment of the linkage procedure, using validated data as a gold standard, are presented in Table 1, for each year.

Sensitivity is most often higher than 90% (97.1% in 2006). Of course the figure regarding the first year is lower. The year 2004 may be considered as an outlier: several hospitals had to change their software and faced many difficulties in collecting data. Specificity is nearly always higher than 85%, except in 2006 (77.7%).

The estimation of the false positive and false negative rates, given in Table 1, also indicates an unexpected higher rate of false positives in 2006 (5.3%). The analysis of the false positives is presented in Table 2, which provides, in case of false positives, the percentage of missing data for each identification item. We can observe that the main reason for false positives appears to be related to missing data; this was particularly the case in 2006. Among false positives, 85.11% were due to perfect concordance between the record of the baby and the record of the mother on the birth dates of the mother and on the zip codes of the main residence of the mother, while the other items were nearly all missing.

Of course, in these cases, agreement between the two records on the 6 identification items cannot be considered perfect. In fact, in case of missing data on an identification item, the two records are considered discordant on this item.

For false negatives, the same analysis is presented in Table 3. Here again, missing data is the main cause. In particular, because of missing data on the maiden and first names of the mother, the two records were often considered discordant on these items, which prevented linkage of these records. Hopefully these errors have been corrected thanks to the validation procedure.

## 4. Discussion

### 4.1. Discussion of the Results

Optimal assessment of perinatal care needs a linkage procedure between successive files for the same patient and between files for the mothers and their corresponding newborn(s). The latter linkage was found to be essential in the assessment of the postnatal consequences of antenatal risk factors and maternal diseases. For instance, in 1999, one maternity hospital showed significantly higher rates of both Caesarean section and neonatal hospitalisation compared with regional and national rates. The linkage procedure revealed that the excess in neonatal hospitalisations was related to an excess in the use of caesarean section in a population of mothers similar to that admitted to other maternity hospitals in Burgundy. This study was performed after adjustment for gestational age and maternal diseases. This discrepancy was particularly noticeable for full-term infants. This finding was a strong argument for reorganising perinatal care leading to a significant decrease in the use of Caesarean section in this hospital.

The fact that each mother corresponded to a newborn and vice versa was particularly helpful in testing the linkage procedure. Indeed, this study demonstrated that coupling the direct linkage of anonymous data files with the validation procedure that takes into account the potential links revealed by the statistical procedure generated very satisfactory results on a regional scale.

This linkage method could also be used to link the siblings (having the same mother). The linkage of

1. *full brothers or sisters* by sorting the dates of birth of all the individuals with the same parents,
2. *half brothers and sisters* by sorting the dates of birth of all the individuals with the same father or the same mother,

would require the implementation of a new identifier, such as the family-based identifier (also including the names and date of birth of the father) [17, 18].

The assessment method used in Burgundy relied on the PMSI system. The main advantages of using the PMSI information system were that this data collection system is mandatory in all French hospitals and that the classification used in this program (i.e., the International Disease classification—10th revision) contained most of the items necessary for an audit of the perinatal network. Moreover, the number of additional items included was limited. Although PMSI was not designed for the assessment of care networks, using this information system made it unnecessary to set up another data collection system in each participating hospital and thus avoided duplication of work.

However, the collection of additional data and the need to extract items from PMSI required extension of the existing software in each hospital. Moreover, these changes involved several companies that were more or less interested in modifying their software. These changes have thus been implemented slowly over a 3-year period.

Overall, the collection and analysis of perinatal data had a substantial cost. Each hospital spent 1000 euros for changes in the PMSI software. Moreover, health professionals and a computer engineer were needed for validation procedures, management of the regional database, statistical analyses, transmitting regional data to the medical steering committee, drafting reports, and helping the hospitals to improve item recording.

Our results showed that satisfactory collection of the linkage items was more difficult to obtain than was the collection of medical items. This is easily explained by the fact that physicians are less motivated to collect administrative data. Therefore, the validation procedure for the identification items was made a cornerstone in the quality process.

The results regarding the assessment of the linkage procedure revealed that sensitivity and specificity are quite high (resp., higher than 90% and 85%). The decrease in sensitivity in 2004 is related to software changes in several hospitals. The decrease in specificity in 2006, as well as the overall false positive rate among the whole period, seemed to be the consequence of missing data. These observations led us to propose harmonization of the procedures (software and rules for collecting data) in all hospitals. This initiative, called the EXTRANAT project, is described in the next section.

### 4.2. Perspectives for the Development of the Perinatal Network

The Burgundy perinatal network was created in 1992 because of the high perinatal morbidity and mortality in France (7th highest among OCDE countries) notably in Burgundy (14th highest in France).

Since 1995, it has included the following.

- A hierarchal interestablishment network of all of the maternity clinics (1 type III maternity clinic, 2 type II maternity clinics, 8 type I maternity clinics, and 4 local perinatal care centers). The missions of each establishment and the criteria and procedures for the transfer of the mother (before or after delivery) and the newborn have been defined.
- A common paper-based record document.
- An original and exhaustive evaluation system, based on the PMSI, for all of the 18 000 annual pregnancies, with anonymization of data in each establishment after discharge, and linkage between the mother and her infant.

Thanks to the combination of these three elements and to the perseverance of those involved in perinatal care and the Burgundy DIM, since 1999, our region has climbed to be among the top three in France with regard to low maternal and perinatal mortality and morbidity.

However, though retrospective analysis of records on perinatal mortality showed a considerable improvement in the quality of intra- and interhospital care between 1996 and 2003 (in-utero transfers for extremely premature births, high-risk patients, antenatal corticotherapy, pediatric care at the maternity clinic), it also revealed a significant deterioration in preclinic conditions, showing the need to set up an “extranet” tool for all of those involved in perinatal care (GPs, specialists, midwives), whether they are based in public hospitals, in private practice or in Mother and Child Care Centres (Protection Maternelle et Infantile).

This is the rationale behind the “Extranat Bourgogne” project, a computer system to facilitate

- the communication (between professionals),
- the training and the evaluation of professional practices (discussions about protocols),
- the implementation of shared interactive computerized medical records, with the medical and organizational references of the network.

Thanks to Extranat, it will be possible to have real-time communication, and regular meetings in the form of videoconferences (telemedicine) to study problem cases. The telemedicine system is already up and running and Extranat is currently being set up.

The aim of this project is to fulfill the three missions of the perinatal network as follows:

- improvement of the quality of care by enabling the health-care professional to input initial medical data (no longer final data at discharge from the establishment). This is a criterion in the improvement in information collection and in the sharing of medical data with complementary specialists (specialists in women's health, specialists in neonatal health for infants that may be transferred to other medical departments or other establishments),
- evaluation of professional practices, which requires identification and anonymization of the professionals concerned,
- evaluation of the medical and organizational aspects of the Burgundy perinatal network (anonymized database with mother-child linkage), already operational and presented in this article.

It is therefore essential for the new Extranat system to use the same patient identification and anonymization system (mothers, newborns, fathers) as the perinatal network, so as to

- provide access (limited and controlled) to medical data for professionals who are treating the mother or newborn (data identified at the physician's practice and/or the medical establishment),
- respect the anonymization requirements for data for the patients (as requested by the CNIL), and for the professionals, in the regional data base.

## 5. Conclusion

It is possible to set up a continuous and exhaustive recording system for linked perinatal data to assess the quality of care on a regional scale. A pre-existing database like PMSI may be valuable, but may have to be extended if necessary. The linkage of anonymous files may greatly enhance the accuracy of the assessment procedure. These principles of assessment of a perinatal network could be extended to other medical domains.

## Figures and Tables

**Table 1:** Assessment of the linkage procedure: sensitivity, specificity, rates of true positives (TP), true negatives (TN), false negatives (FN), and false positives (FP), for each year.

Years	Records no.	Sensitivity (%)	Specificity (%)	TP (%)	TN (%)	FN (%)	FP (%)
1998	17865	89.04	95.25	85.68	3.59	10.55	0.18
1999	15769	97.53	97.82	89.04	8.52	2.25	0.19
2000	20941	97.24	97.77	86.35	10.50	2.45	0.70
2001	25070	93.03	87.47	78.99	13.20	5.92	1.89
2002	25489	94.17	89.74	79.72	13.76	4.94	1.57
2003	25264	93.82	86.84	78.64	14.05	5.18	2.13
2004	25976	88.26	87.31	69.14	18.91	9.20	2.75
2005	24440	93.13	86.02	70.57	20.84	5.20	3.39
2006	27238	97.09	77.74	73.97	18.51	2.21	5.30

**Table 2:** Percentages of missing data for each identification item, among false positives, for each year.

Years	Maiden name (%)	Mother's first name (%)	Mother's date of birth (%)	Infant's first name (%)	Infant's date of birth (%)	Zip code (%)
1998	43.75	18.75	18.75	46.88	40.63	18.75
1999	33.33	33.33	20.00	56.67	53.33	20.00
2000	3.71	1.68	1.89	22.46	16.72	1.33
2001	89.87	90.08	0.21	97.89	97.68	0.21
2002	78.30	79.05	29.68	97.01	96.51	0.50
2003	84.94	84.39	0.37	98.14	98.14	0.37
2004	69.33	56.30	0.14	86.41	95.38	0.00
2005	82.49	58.82	8.70	74.76	79.11	8.82
2006	75.21	71.05	0.07	97.02	97.85	0.14

**Table 3:** Percentages of missing data for each identification item, among false negatives, for each year.

Years	Maiden name (%)	Mother's first name (%)	Mother's date of birth (%)	Infant's first name (%)	Infant's date of birth (%)	Zip code (%)
1998	95.33	97.40	97.40	91.14	90.98	0.32
1999	55.49	94.08	94.08	12.68	5.63	2.54
2000	62.62	67.91	69.28	20.94	16.05	25.24
2001	45.04	40.53	23.74	27.92	27.11	2.63
2002	58.22	27.40	29.47	34.47	19.30	1.03
2003	33.10	32.42	39.53	59.56	24.92	1.91
2004	27.46	25.66	11.85	38.80	30.85	0.63
2005	88.36	87.66	19.65	87.19	81.92	3.07
2006	74.96	74.46	71.97	47.43	32.34	1.00
